# Therapeutic and Formulation Innovations in the Management of Canine Otitis Externa

**DOI:** 10.3390/pharmaceutics17101332

**Published:** 2025-10-14

**Authors:** Yunmei Song, Sangseo Kim, Songhita Mukhopadhyay, Souha H. Youssef, Jin Quan Eugene Tan, Emily Josephine Weir, Stephen W. Page, Sanjay Garg

**Affiliations:** 1 Centre for Pharmaceutical Innovation (CPI), College of Health Sciences, Adelaide University, Adelaide, SA 5000, Australia; may.song@unisa.edu.au (Y.S.); sangseo.kim@mymail.unisa.edu.au (S.K.); songhita.mukhopadhyay@mymail.unisa.edu.au (S.M.); souha.youssef@unisa.edu.au (S.H.Y.); eugene.tan@sa.gov.au (J.Q.E.T.);; 2 Neoculi Pty Ltd., Burwood, VIC 3125, Australia; swp@advet.com.au

**Keywords:** canine otitis externa, diagnosis, formulation development

## Abstract

Canine Otitis Externa (COE) is a challenging otological disorder in dogs which causes significant irritation and discomfort. This comprehensive review provides an extensive analysis of COE with emphasis on the fundamentals of the condition, starting with the basic anatomy of the canine external ear canal, followed by pathophysiology and diagnosis of COE. Furthermore, novel therapeutic interventions, formulation considerations, and challenges, with the perspective of future directions in the field of COE management, are described. Diagnostic models, including clinical examination, cytology, and susceptibility tests, are presented to provide an overview of the processes involved in detecting and selecting appropriate therapies for the management of COE. Moreover, this paper describes the limitations of current therapies with considerations for the selection of alternative novel treatments such as aromatherapy, acupuncture, bacteriophages, nutraceuticals, and nanomedicines. This review places particular emphasis on the pharmaceutical formulation of topical products used for COE treatment. Various factors, including osmotic pressure, safety profile, viscosity, bioadhesion, and formulation pH, must be considered when developing topical preparations. These parameters are critical in formulation development to enhance therapeutic outcomes and minimise potential side effects. Finally, potential advancements in COE management are highlighted, including the integration of microbial genomics and the significance of managing the microbiota. Overall, this review serves as a valuable resource for those interested in the future of topical treatment of COE by providing a deep understanding of diagnostic, therapeutic, and medical interventions for effective management.

## 1. Introduction

COE is an inflammatory condition of the outer ear canal that affects about 5–20% of dogs globally and 30–40% in tropical environments [1]. In Kumar’s study, COE case incidence peaked in August (28.33%) and October (26.66%), with the lowest in December (10.00%) in the Jammu region, India [2]. The clinical manifestations of COE include erythema, pruritus, swelling, skin itching, and discharge from the external ear [3]. The magnitude of infection is determined by several factors, including age, sex, conformation of the ears, pH of the external auditory canal and distribution of hair follicles, breed, exposure to water, and trauma to the ear canal, inter alia [4,5].

The treatment of COE generally requires a comprehensive approach considering applicable risk factors, including primary and secondary causes, as well as predisposing (altering the ear canal environment, raising the risk of otitis externa) and perpetuating factors (worsening and maintaining ear disease, even after the primary cause is treated) [6,7]. Treatment generally involves cleaning the external ear canal and the use of topical antimicrobials and corticosteroids, which can be delivered to the ear canal via ear drops or gels [7,8]. However, drug delivery to the ear canal can be challenging due to the complex anatomy of the canine ear and the difficulty accessing the affected area [8], and potential ototoxicity if the tympanic membrane is perforated [8].

While there is no universally accepted safe upper limit for topical otic drugs in dogs; safety must be interpreted on individually. For example, experimental studies have shown topical gentamicin at approximately 3 mg/mL [9] and chlorhexidine acetate 0.2% [10] were tolerated in dogs under the tested regimens.

Clinical experience supports the safety of several topical antimicrobials when applied to canine ears. Stephen White presented safety data in Proceedings of the Central Veterinary Conference for some topical medications used, namely enrofloxacin at 4–10 mg/mL in combination with Tris-EDTA has been reported to be well tolerated in middle-ear use without apparent ototoxicity, and the marketed product Baytril Otic (5 mg/mL enrofloxacin with 10 mg/mL silver sulfadiazine) is likewise considered safe. On the other hand, polymyxin B is potentially ototoxic and contraindicated if the tympanum is perforated, and documented cases of ototoxicity have been reported with ticarcillin–clavulanate. Aminoglycosides such as amikacin (50 mg/mL) and tobramycin are occasionally used topically, but they carry a recognized risk of ototoxicity if middle-ear exposure occurs [11].

In recent years, a range of different strategies has been suggested for the treatment of COE, including identifying new drug entities as well as novel clinical interventions to improve the efficacy of treatment and minimise potential adverse effects. These novel interventions include topical aromatherapies [12], bacteriophage therapy [13], nutraceuticals [3], and acupuncture [14]. However, further research with more extensive in vivo clinical data is required to establish their efficacy and safety profiles before seeing clinical translation [15].

Moreover, otic drug delivery systems have gained significant interest and experienced rapid advancements over the recent decades [16]. This has led to the development of new topical otic formulations and new drug delivery platforms such as nanomedicines [17]. Such novel formulations can effectively provide the active ingredient to the affected area in order to maximise the therapeutic potential whilst minimising systemic exposure [16].

This review provides an overview of COE, the challenges associated with current treatment strategies, and the potential benefits and limitations of these interventions. To our knowledge, this narrative review for the first time presents insights into the key considerations for the development of novel topical formulations for the management of COE.

## 2. Basic Anatomy of the Canine External Ear

**Figure 1:**
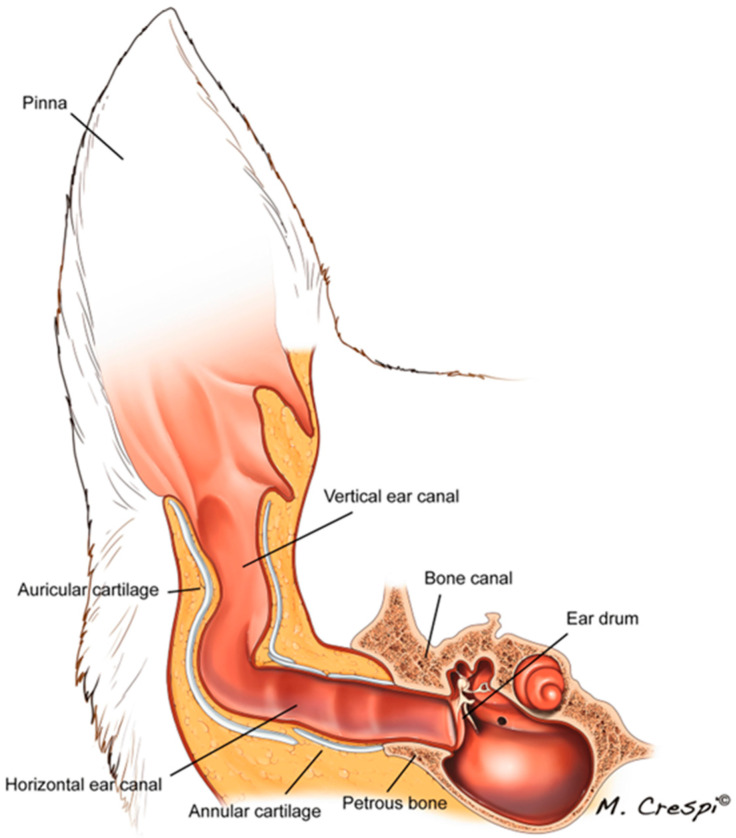
A schematic diagram of the canine ear. Reprint with permission from [18]; copyright permission license provided by Springer Nature and Copyright Clearance Center 2024.

The canine ear can be anatomically categorised into four separate components: the pinna, external ear canal, middle ear, and inner ear (Figure 1) [18]. In this review, the focus will be mainly on the external part of the ear, the pinnae, and the external ear canal within the context of COE.

The anatomy and physiology of the canine ear have been comprehensively described by Cole [19], with a selected summary following. The pinnae are the visible portion of the external ear, which is the first part of the ear that comes in contact with sound waves [19]. It plays an essential role in funnelling sound waves to the tympanic membrane. Depending on the breed of the dog, the pinna can either be in an erect or pendulous conformation. The pinnae consist primarily of auricular cartilage, and both concave and convex sides of the pinnae are layered with sebaceous glands, apocrine glands, and hair follicles. The number of hair follicles is significantly higher on the convex side than on the concave. Ear canal hairiness and a pendulous ear conformation facilitate heat and moisture retention, creating a favourable environment for microorganisms to proliferate [19]. The canine external ear canal consists of two elastic cartilages, the auricular and annular cartilages. The tragus, a quadrangular plate of cartilage, forms the lateral boundary of the external ear canal that lies opposite the anthelix, a low transverse ridge present on the medial wall of the ear canal. An anatomic region on the external ear, termed the intertragic incisure, can be used to guide an otoscopic cone or oto-endoscope in the ear canal for examination purposes. There is about a 45–90-degree bend around the medial region of the ear canal, forming the vertical and horizontal ear canal. Separating the vertical and horizontal ear canal is a large cartilage ridge, which makes it hard for otic examination to be conducted on the horizontal ear canal. This ridge can be elevated by lifting the ear pinna to improve access into the horizontal ear canal [19]. The external ear canal of dogs is layered with stratified squamous epithelium containing hair follicles, and sebaceous and apocrine glands that produce ear wax (cerumen). This production is valuable in trapping debris and has other antimicrobial properties. The normal flora of the ear canal includes bacteria such as *Staphylococcus*, *Bacillus* sp., *Corynebacterium* sp., *Streptococcus* sp., and *Micrococcus* sp., and yeasts. The structure of the ear canal is similar to the pinna, containing less hair; sebaceous and ceruminous glands are also present. These sebaceous glands are highly prominent in the distal parts; their duct open into hair follicles of the ear canal. Ceruminous glands, resembling apocrine sweat glands, are situated deeper within the dermis. These glands, more abundant in the lower third of the ear canal, also open into hair follicles or directly onto the ear canal surface. There is variation between breeds concerning the size and structure of these glands, with long-haired breeds having more glandular tissue [19].

It has been observed that dogs with COE show an increasing amount of glandular tissue compared to normal dogs [20]. Increased cerumen production and heightened cerumen gland activity are common features in otitis-suffering dogs [20]. Whilst the mean pH of the normal external ear canal has been reported to be around 6.1 in males and 6.2 in females, a rise in pH and relative humidity (microclimate of the external ear canal, normal dogs-88.5%) has been observed in the ear canal of dogs with COE [20].

## 3. Canine Otitis Externa

COE is a common infection of the external ear, and its signs include erythema, itching, swelling, and discharge from the external ear [3]. In some cases, dogs may also develop hearing loss [21,22]. The causes of COE are often multifactorial and can be categorised into primary, secondary, predisposing, and perpetuating factors [6,23].

Primary causes are events or diseases that are directly responsible for the initiation of the disease process in a healthy ear, including hypersensitivity, endocrine disorders, autoimmune conditions, atopic dermatitis, keratinisation disorders, and foreign bodies [4,6]. These factors should be addressed carefully when developing treatment plans to prevent potential disease recurrence and progression to chronic otitis externa [6]. Secondary causes of COE are factors that can exacerbate or perpetuate an existing condition [24]. Secondary causes are often due to microbiological infection, including bacterial, fungal, and parasitic infection [25]. Bacterial species such as *E. coli, Staphylococcus* spp., *Proteus* spp., or *Pseudomonas* spp are most commonly found, together with the commensal yeast, *Malassezia pachydermatis* [25,26].

Predisposing factors, including body weight, sex, age, ear shape/conformation (i.e., pendulous or erect), hairiness, breed, and humidity, generally do not cause COE by themselves but may increase its prevalence [21]. It has been found that dogs older than 5 years are more susceptible to COE, potentially owing to immunosuppression, increased sebum production, and inadequate hygiene [27]. Male dogs experience a higher incidence of COE as they have higher levels of androgen production that can stimulate the production of sebum, which is a known risk factor for COE [27]. Canine breeds with pendulous ears have higher incidence rates compared to breeds with erect ears. [4,26]. In addition, breeds with ear canals replete with hair experience a higher incidence [4,24]. Climate is another risk factor. A study conducted in India demonstrated that the prevalence of COE is more evident in the summer when a high level of humidity provides an ideal environment for the growth and development of bacteria and yeast, leading to an increased risk of COE [27,28].

If these factors are not identified and managed promptly, the inflammatory processes may progressively damage the ear canal, leading to perpetuating changes over time and preventing healing. Inadequate or delayed management of underlying factors and recurrent infections may increase the risk of irreversible pathological changes. For example, whilst nodular hyperplasia of the epidermis and glands gives the ear canals a distinct cobblestone appearance, further thickening of the epidermis and dermis can occur, accompanied by ear canal stenosis, occlusion, fibrosis, and mineralisation. These changes can ultimately lead to complications such as tympanic membrane rupture, otitis media, and cholesteatoma formation [29]. This reinforces the early diagnosis and intervention of such factors to prevent COE from progressing to its chronic state.

### 3.1. Diagnosis of Canine Otitis Externa

Diagnosis of COE often requires a systematic approach. A physical and otic examination should be performed, followed by otoscopy and a cytological evaluation [6].

Otoscopy allows for an internal assessment of the ear canal and identifies any complicating issues in dogs with COE. During this procedure, the diameter of the ear canal can be assessed, and any ulcers, exudate, parasites, lesions, or damage to the tympanic membrane can be identified [30]. In the presence of exudate and debris, cytology is recommended in order to help elucidate microbial causes [30].

Cytology involves the microscopic examination of cells recovered from tissue or fluid [30]. In the case of COE, a sample of exudate or debris from the ear canal can be stained (e.g., Giemsa) to identify the characteristics of presenting organisms (yeasts, rods, cocci), which can then be used to predict susceptibility to specific antimicrobials based on historical data. It helps to select an effective anti-microbial agent with a narrow spectrum, taking into account the identified organisms and their expected antimicrobial susceptibility profile [30,31]. Nonetheless, the diagnostic accuracy of cytology is influenced by operator expertise and clinical context. For example, a large veterinary study reported sensitivities ranging from 38–76% (depending on the criteria for agreement with histology) and a specificity of approximately 83% for neoplastic lesions [32]. Similarly, another study using the International System for Reporting Serous Fluid Cytology, effusion cytology showed a sensitivity of 60% and a specificity of 99% when correlated with histology [33]. These findings highlight that diagnostic reliability may be lower in general practice compared with specialist dermatology settings, where sampling and interpretation are more standardised.

### 3.2. Medical Management of Canine Otitis Externa

The initial step of COE treatment often involves the cleaning of the external ear canal. Ear cleaning is recommended as it removes debris, exudate, and microbes from the canal [7].

Topical treatment is the main route of administration for COE, with topical or systemic glucocorticoids administered initially to reduce associated pain and swelling, thus facilitating animal handling for ear cleaning and drug administration. Additionally, glucocorticoids prevent biofilm development and consequent chronic otic changes [6].

Antibiotic treatment for topical administration can be based on the cytology results, with agents selected based on local experience [34]. It is common for such antibiotics to be combined with a corticosteroid and antifungal agent (in the case of *Malassezia* infection) [8]. Such a combination helps in the effective management of the condition. However, systemic administration may be warranted if chronic or severe COE requires long-term use of oral antibiotics to effectively manage the infection or when there are issues with compliance or local adverse reactions to topical therapy [8].

Especially when otitis is associated with atopic dermatitis, anti-inflammatory, and immune-modulating compounds such as ciclosporin, glucocorticoids, lokivetmab, and oclacitinib may be indicated [35,36,37,38,39]. For managing recurrent COE, topical or systemic glucocorticoids are preferred for reversing chronic pathological changes therapy, while ciclosporin may be useful for continuing care. However, lokivetmab and oclacitinib are considered to have limited effectiveness for the treatment of reversing chronic pathological changes [29].

Current therapies for COE can be effective; however, there are several limitations and potential drawbacks. Overall, topical treatment is the preferred choice and is able to manage most cases of acute otitis externa; additionally, a broad variety of available anti-inflammatory and anti-microbial medicines offer great options [8]. However, it is important to select the right medications and demonstrate their administration to dog owners to ensure high levels of compliance, especially when long-term use is necessary [8].

Comprehensive guidance on the selection of antimicrobial agents and management of COE is provided by the guidelines of the Australasian Infectious Diseases Advisory Panel (AIDAP) [40], and by Guidance for the Rational Use of Antimicrobials—Recommendations for Dogs and Cats (GRAM) [41] with additional non-antimicrobial guidance outlined by Nuttall [8,29] and by Harvey and Paterson [20].

### 3.3. Alternative and Novel Therapies for the Treatment of Canine Otitis Externa

**Figure 2:**
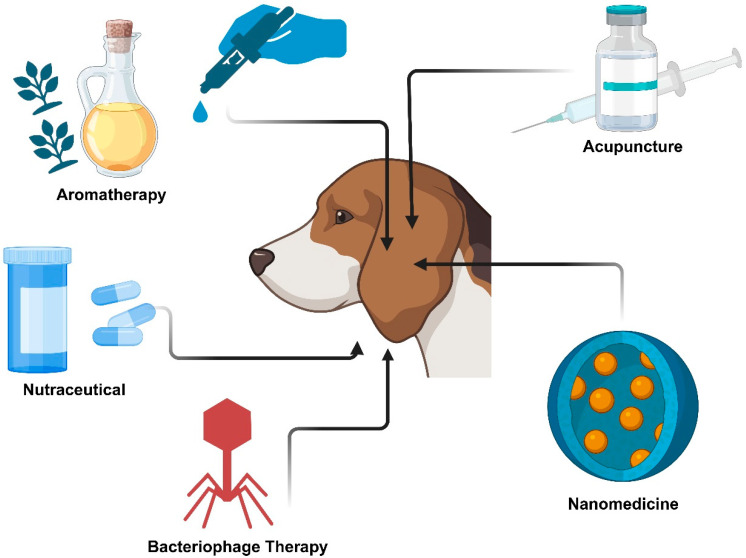
Representation of alternative and novel therapies for canine otitis externa. “Created using Biorender https://app.biorender.com/, accessed on 8 July 2025”.

As the predisposing and perpetuating factors are not well managed and poor compliance is frequently associated with current treatments for COE, alternative and novel therapies have been explored as potential solutions. These therapies include aromatherapy [12], acupuncture [14], bacteriophage therapy [13], and nutraceutical [3] as well as novel drug delivery systems such as nanomedicine [17] (Table 1, Figure 2).

Firstly, aromatherapy involves the use of volatile essential oils administered by diffusion, nebulisation, massage, or topical application to produce a therapeutic response [12]. The efficacy of aromatherapy was evaluated in 11 dogs with COE [12]. The control group (five dogs) received topical amoxicillin-clavulanic acid, ciprofloxacin, and ketoconazole preparations, whilst the treatment group (6 dogs) received topical aromatherapy with base oil, bergamot, lavender, tea tree oil, and roman chamomile, applied to the ear canal twice daily for two weeks. This study’s results demonstrated that the colony counts of bacterial cells in the treatment group were significantly lower than the control group. Therefore, the antibacterial activity may support the treatment of COE with aromatherapy [12]. The two most important oils, oregano oil and thyme oil, previously demonstrated promising activity against all the strains of bacteria and fungi isolated from canine cases of COE, including *Staphylococcus pseudintermedius*, *β-haemolytic Streptococcus* spp., *Pseudomonas aeruginosa*, *Proteus mirabilis,* and *Malassezia pachydermatis* [42].

A form of chemical acupuncture is apipuncture, wherein diluted bee venom is conventionally used in the relief of pain and inflammation [43]. It can be administered with topical aromatherapy, and this new approach has shown promising preliminary results in treating Malassezia-related COE [14]. The efficacy of the combination of aromatherapy and apipuncture was compared to ketoconazole in 10 dogs in a clinical study [14]. The treatment group was treated with aroma oil containing tea tree oil, bergamot oil, and sweet almond oil, as well as a bilateral injection into the 2I-19 (ting gong acupoint) of apitoxin diluted with saline and mixed with lidocaine HCl [14]. Clinical scores of pruritus, redness, cerumen, odour, and the number of yeasts were evaluated to test for efficacy, whilst serum levels of alanine transferase were tested for hepatotoxicity [14]. The results of this small study demonstrated that the combination therapy was not only as effective as the treatment with ketoconazole but also demonstrated a better safety profile [14].

Bacteriophages are viruses that can selectively kill bacteria without affecting human or animal cells [13]. Bacteriophage therapy has proven to be effective against *Pseudomonas aeruginosa,* which is a common multi-drug-resistant bacterial species, typically requiring broad-spectrum antibiotic treatment [13,44]. The effectiveness of a topical bacteriophage mixture containing 6 different bacteriophages was demonstrated against multiple *Pseudomonas aeruginosa* strains in a study involving 10 dogs over 48 h. The *Pseudomonas aeruginosa* counts from aural swabs were significantly reduced, and visible clinical improvements were observed after topical application. Additionally, the canine test subjects had no signs of local, immunogenic, or systemic toxicity reactions after treatment with bacteriophage therapy [13].

Nutraceuticals have also shown clinical benefits in alleviating signs of chronic bilateral COE, such as ear canal occlusion, erythema, discharge, and odour when used in conjunction with a standard topical treatment [3]. A randomised controlled clinical trial involving 30 dogs with chronic COE evaluated the effects of an anti-inflammatory and antioxidant diet containing a combination of fish hydrolysed proteins, rice carbohydrates, tea tree (*Melaleuca alternifolia*), linden tree (*Tilia cordata*), garlic (*Allium sativum* L.), dog rose (*Rosa canina* L.), zinc, and omega 3 and 6 ratios (1:0.8). After a 90-day intervention, the treatment group demonstrated a significant decrease in the mean COE score intensity of all signs (*p*  <  0.0001), compared with the control group that received a standard diet with topical therapy, except for infected by *Malassezia pachydermatis,* in which only a slight reduction was observed [3].

A novel drug delivery platform utilising nanotechnology has shown promising results in COE treatment. In a recent study, the antibacterial effects of silver nanoparticles have been explored in vitro against *Staphylococcus pseudintermedius*, a primary pathogen associated with COE [17]. Ten isolates of *S. pseudintermedius* obtained from subjects with COE were treated with silver nanoparticles, and the antibiofilm activity was evaluated with Congo red agar (CRA) methods using a modified microtiter plate and scanning electron microscopy [17]. The nanoparticles exhibited a significant dose-dependent antibiofilm response; hence, a reduction in biofilm formation was observed at concentrations of 10 and 20 µg/mL with a *p*-value of less than 0.05. *S. pseudintermedius* treated with 20 µg/mL of silver nanoparticles also showed reduced bacterial slime formation compared to the control group on CRA plates [17]. Whilst this research highlights the enhanced antibacterial potential of silver nanoparticles, it is important to note the limitations of in vitro conditions. As the canine ear represents a complex and dynamic microenvironment, it may not be fully replicated in laboratory conditions. Translation into clinical practice would require addressing key challenges such as formulation stability, local tolerability, pharmacokinetics, and the potential toxicity of silver nanoparticles in dogs. Thus, further in vivo studies are essential to validate their therapeutic potential and safety in COE management.

However, the ototoxicity risk of nanoparticle therapies requires careful evaluation before clinical application. Studies performed on rats caused significant, dose-dependent alterations in the permeability of the middle ear mucosa of biological barriers, external ear canal skin, and the inner ear [45]. Another study showed that combined exposures of AgNPs and noise caused permanent damage to the hair cells that are responsible for high-frequency perception [46]. On the other hand, other reviews of nanoparticle strategies for inner-ear delivery discussed that many nanoparticle platforms (particularly when fabricated to reduce oxidative stress or encapsulate active agents) demonstrated biocompatibility in vivo, yet further otic safety tests must be performed [47].

Alternative and novel therapies have been explored as potential solutions to the limitations of current treatments for COE. However, to reflect the requirements of evidence-based dermatology [15], further research is required to establish the efficacy and safety profiles of these therapies for clinical use. In addition to exploring alternative treatments, it is also crucial to improve current topical formulations. An essential aspect of the management of COE is to ensure that the formulation delivers the active ingredients to the lesioned area in a manner that maximises therapeutic potential whilst minimising local and systemic adverse effects. Furthermore, the formulation and its delivery device should be easy to use and comfortable for the recipient animal.

## 4. Considerations for Otic Formulation Development

Topical drug delivery, including drops, gels, creams, ointments, and foams, is commonly used for the treatment of COE, allowing for medications to be delivered locally into the external ear canal. [16]. This route of administration helps to achieve high local drug concentrations, which is often necessary for effective treatment, whilst minimising unnecessary systemic exposure and the development of bacterial resistance [48].

The effectiveness of topical formulations for COE is influenced by many factors, including the physicochemical properties of the active ingredient, characteristics of the formulation, and its ability to deliver drugs to the target site at an effective concentration for an intended retention time [23]. Selecting a drug candidate for topical applications with suitable physicochemical properties and skin permeability to treat COE is crucial. However, it is also important to consider other factors such as the site of action, i.e., within the ear canal and epidermis, bioadhesion to the target site, formulation pH, viscosity, spreadability, and the choice of application devices. These considerations ensure adequate retention time, ease of administration, and appropriate coverage of affected areas [23]. Guidelines on the development of otitis products have been published by the US FDA [49].

The physicochemical properties of the active ingredients, formulation design, and excipients are key parameters impacting the likelihood of achieving the desired target product profile [50]. In this context, our previous research investigated the effect of various excipients (aqueous and non-aqueous systems) on the basic properties of otic formulations [23]. Out of nine selected marketed otic products used for the treatment of COE in Australia, eight (Surolan^®^, Derm Otic^®^, Mometamax^®^, Easotic^®^, Canaural^®^, Osurnia^®^, Aurizon Ear Drops^®^, and Apex PMP^®^) are nonaqueous systems, whilst only Baytril Otic^®^ is an emulsion system [23]. Otic formulations are generally in the form of solutions, suspensions and drops, and include many excipients such as pH modifiers (e.g., citric acid), antimicrobial preservatives (e.g., aluminium acetate), suspending agents (e.g., hydroxyethyl cellulose), stabilising agent/thickening agent (e.g., Povidone K30), emollient (e.g., polyoxyl 40 stearate), solubilising agent/wetting agent (e.g., polysorbate 20) and tonicity agent (e.g., sodium chloride) [16]. Recent otitis products approved for use in the US include DuOtic^®^, authorised by the US FDA in March 2024 [51], and Mometamax Ultra^®^ approved by APVMA [52], both of these are suspensions providing excellent activity against COE.

### 4.1. Physicochemical Properties of Active Ingredients

The successful treatment of COE requires effective deposition of drugs at the site of infection with superficial skin layer penetration; however, in most instances, delivery and uptake are impeded by the outermost skin barrier known as the stratum corneum. Superficial and transdermal delivery depends on many factors, such as the physicochemical characteristics of the drug itself, as well as on the excipients incorporated into the formulation [16]. Molecular weight (<500 Da), partition coefficient (log *P,* between 0 and 5), low melting point, and ionisation are the key physicochemical parameters to consider for effective skin permeation [53,54]. Generally, non-ionised forms of drugs are preferred over ionised forms [55]. The molecular weight and XLogP3 values (lipid-water partition coefficient) of several drugs that are currently used in the treatment of COE on the market in Australia or the UK are summarised in Table 2.

### 4.2. Safety Profiles

The selection of a new drug candidate for inclusion in otic or systemic formulations should be carefully considered based on its safety and toxicity profile. For example, fluoroquinolones are one of the most versatile antimicrobial agents, as they are highly effective against numerous Gram-positive and Gram-negative pathogens [57]. However, fluoroquinolones should not be administered systemically to young and rapidly growing dogs because fluoroquinolones can cause a non-inflammatory, erosive form of arthropathy [58]. Furthermore, several other antibiotics are potentially ototoxic if they gain access via a ruptured tympanic membrane to expose the cochlear or vestibular nerves to toxic concentrations [59]. As ear trauma or COE can compromise the barrier function of the tympanic membrane, the use of ototoxic antibiotics such as polymyxin B and neomycin should only be administered with extreme care [8,60].

### 4.3. Osmotic Pressure

Whilst the action of COE treatment should ideally be localised to the application site with minimum systemic exposure [23], skin permeability to many drugs is generally governed by the outermost layer called the stratum corneum [61]. Although the role of osmotic gradients in facilitating skin permeation is well-supported in human skin studies, similar investigations have not been conducted in canine skin. Based on the human skin studies, there exists a large osmotic gradient across the skin that can be measured as a difference in solute concentrations between either side of a semipermeable membrane, which may serve as a driving force for drug partitioning into the skin [62,63]. However, applying a topical formulation to the skin may change the osmotic gradient of the skin substantially, in turn, affecting the degree of drug permeation [64]. External water gradient was shown to regulate the movement of drugs across the skin, as demonstrated in the topical application of metronidazole and methyl salicylate with different water chemical potentials. The study demonstrated that a low osmotic gradient, leading to high SC hydration, drastically increased the drug permeation through the skin. In general, the osmotic pressure of a topical formulation can be altered by varying the concentration of sodium chloride to achieve the desired target profile [64].

### 4.4. Bioadhesion

Bioadhesion is important in topical drug delivery systems to ensure the application materials remain in contact with the biological surface for the entire duration of application [65]. Good bioadhesive properties are desirable as they facilitate the targeted and sustained release of the drug by increasing the drug’s residence time [65]. Formulations with good bioadhesion can also improve treatment compliance and minimise drug loss from the site of infection [23]. In general, factors such as polymer type, environmental temperature, and pH may influence the bioadhesion of otic formulations [5,23].

In general, polymers are added to modify various properties like bioadhesion, viscosity, and controlled release of drugs from the formulation. Polymer selection is an important criterion because it may affect the nature of the bioadhesive property of the formulation. For example, Osurnia^®^ contains hydroxypropyl methylcellulose, a non-ionic polymer whose bioadhesion was found to be higher compared to the other eight otic products studied; this is because it can swell to a higher level with consequent development of a higher degree of bioadhesion [23]. However, the viscosity of the pH-responsive polymers—for instance, cationic polymers like chitosan, or anionic polymers like sodium alginate and carboxymethyl cellulose—is considerably influenced by changes in the pH of the surrounding microenvironment [66]. which may affect their bioadhesion. For example, chitosan, as a cationic polymer, exhibits strong adhesion at an acidic pH due to protonated amine groups, allowing for strong electrostatic attraction, whereas progressive deprotonation significantly reduces adhesion at higher pH, leaving only weak hydrogen bonding and van der Waals interactions [67]. Such findings highlight the importance of polymer selection in canine otic formulations, as the disease-modified microenvironment might potentially impact the therapeutic performance of the otic formulation. In case of otitis externa, the normal pH of the external auditory canal (~4.6–7.2) may increase, or, in acute conditions, decrease, joined by an increase in humidity and temperature [68]. These modifications affect the surface chemistry of the cerumen, which in turn affects the interaction with topically applied polymers. Polymer-based formulations typically use topical-based hydroxypropyl methyl cellulose (HPMC), which depends on obtaining optimal bioadhesion with prolonged residence time [23]. In diseased-state cerumen, the typical properties of polymers, namely swelling and hydration, which generally promote adhesion on lipid-rich surfaces, are significantly compromised [69]. Furthermore, changes in the pH can affect the ionization state and charge distribution of both cerumen constituents and the polymer molecules, thus affecting the electrostatic interactions, which are critical for adhesion [23].

### 4.5. Viscosity

Viscosity is another key modifiable property for topical preparations, as it can influence drug retention time. Viscosity can generally be modified by adjusting the concentration of polymers within the formulation [70]. Whilst high viscosity is beneficial for promoting product retention on the site of application, it has an inverse relationship to spreadability [23,71]. Low viscosity can reduce the effectiveness of the product due to poor spreadability and limited retention time [72], whilst viscosity can also affect the ease of product dispensing from the packaging [73]. An ideal formulation should exhibit properties where it flows easily when stress is applied to the packaging, but returns to sufficient viscosity once applied topically to maintain effective drug retention time [72,73].

Poloxamers are one of the commonly used polymers in otic formulations as viscosity-enhancing agents that exhibit thermosensitive characteristics desirable for otic application. The poloxamer solution remains fluid at low temperatures, making it easier for otic application whilst undergoing a rapid phase transition and transforming into a gel at the higher temperatures (25 °C ± 1 and 42 °C ± 1) that occur at the target site [74]. Furthermore, hydroxypropyl methylcellulose (HPMC) and sodium carboxymethyl cellulose may also be used in the otic formulations as a viscosity-enhancing agent. A recent study demonstrated that Poloxamer 407 19% (*w*/*v*) and HPMC K100M 0.2% (*w*/*v*) could form an ideal in situ gelling otic formulation [75].

### 4.6. Formulation pH

The pH value of the formulations is also crucial to prevent disruption of the microenvironment of the canine external ear canal epithelium. The normal canine skin has sufficient buffering capacity to maintain its pH between 5.5 and 7.2 [23], which is slightly more alkaline than human skin [23]. In dogs suffering from chronic COE, the pH tends to be on the upper side of the pH range (6.0–7.4), whilst the pH of the ear in dogs suffering from acute COE tends to be on the lower side (5.2–7.2) [19]. Although formulations with extremely acidic or alkaline pH can help to inhibit the growth of microorganisms that contribute to COE, they can also increase the risk of skin irritation, which can potentially lead to poor treatment acceptability [76]. Moreover, the use of acidic ear cleaners can potentially inactivate some commonly used antimicrobials for COE, such as aminoglycosides and fluoroquinolones [8]. Therefore, commercial products, such as Baytril Otic^®^ containing enrofloxacin and silver sulfadiazine, have been formulated in an aqueous solution that has an approximate pH value of 6.26 ± 0.04, which is consistent with the average pH of the ear canal epithelium [8].

### 4.7. Application Devices

A crucial aspect of veterinary drug development is ensuring owner compliance, which is affected by the ease and frequency of product administration [77]. Compliance issues may arise when the medication requires multiple daily doses, has a difficult administering system, or when COE causes distress in the canine [78]. A user-friendly device can make administration easier and more accurate, leading to more reliable dosing and better treatment outcomes. Owner compliance and satisfaction were evaluated using two different topical application products—Easotic^®^, a 10 mL pump dispenser dosed once daily for 5 days; and Surolan^®^, a 15 mL dropper vial applied as 3–5 drops per ear for at least 7 days [78]. The study included 42 dogs of different breeds divided evenly into two groups, with one group receiving Easotic^®^ and the other Surolan^®^ [78]. Dog owners rated the benefits and drawbacks of the products, as well as their satisfaction with the frequency and duration of treatment [78]. Veterinarians evaluated the ease of use of the products and the reliability of owner compliance [78]. Results from this study suggested that owners administering Easotic^®^ were more compliant than those using Surolan^®^ due to the ease of application and less frequent dosing required by Easotic^®^ [78]. Reduced compliance with Surolan^®^ was due to owners forgetting a dose or inaccurately counting the number of drops of product to be administered [78].

Droplet volume is another important parameter that can be controlled by application devices to ensure dosing consistency. A study assessing the uniformity of administration of betamethasone ear drops found that there was a statistically significant difference between male and female volunteers in the doses administered using an ear drop [79]. In order to accurately weigh 0.025 g of betamethasone ear drops, it was found that males, on average, dispensed 4.5 drops, whilst females only dispensed 1.8 drops [79]. Additionally, there was also a high level of intra-volunteer variability, which could potentially lead to dosing inconsistency and poor clinical outcomes [79].

Overall, it is critical to consider the dosing frequency and ease of administration, as well as the design of more user-friendly and accurate application devices in the development of otic products for COE.

## 5. Future Trends of COE Treatment

Global concerns about antimicrobial resistance and its impacts on One Health have led to increased antimicrobial stewardship with a focus on the appropriate selection and use of antimicrobials in companion animals, particularly those agents considered critically important in human medicine [80]. For example, recent studies have found a high frequency of multidrug resistance (including to critically important antimicrobial agents) in *Staphylococcus pseudintermedius* isolated from cases of otitis in dogs from Queensland, Australia [81]. Consequently, the future of COE treatment involves the exploration of new strategies, such as the use of lower-risk antibiotic combinations [82], the discovery of novel antibacterial targets, and the utilisation of alternative treatment measures that can enhance antibiotic treatment, including natural microbiota or probiotics. Current metagenomic studies [83] clearly establish the fact that microbial changes observed in otitis externa, especially depletion of the commensal microbes, generate a rationale for therapies aimed towards microbial restoration [84].

Since the first bacterial genome was sequenced in 1995 [85], noteworthy advancement in microbial genomics has led to the identification of potential novel targets for antibacterial drugs, such as efflux pumps and riboswitches [86]. Efflux pumps, especially in Gram-negative bacteria, play an important role in transporting toxic substances out of bacterial cells and into the external environment. Efflux pumps have become one of the significant causes of antimicrobial resistance in biofilm-embedded bacteria [87]. Different studies have indicated that efflux pump inhibitors are able to combat antimicrobial resistance by increasing internal antibiotic concentrations and decreasing bacterial survival and pathogenic potential [86]. Another approach involves riboswitches, regulatory elements in bacterial mRNA that have a huge role in controlling many functions, like the synthesis and transport of important metabolites, motility regulation, and biofilm formation [86]. The interaction of a specific ligand with the riboswitch finally leads to the repression of gene expression encoded by the associated mRNA [86]. The potential of developing analogues that can bind to and inhibit riboswitches has been explored, although this paper did not detail specific examples of the growth of Gram-positive bacterial species through the repression of specific metabolic pathways [88].

It is also important to address the underlying disturbance in the ear microbiota as part of the treatment plan for COE [89]. A diverse and intricate microbial population on the skin plays an important role in protection, pathophysiology, and disease development [90]. One approach to the prevention of microbial infection involves the manipulation of the benefits provided by microbiota [90]. Thus, a treatment that corrects an imbalance or dysbiosis of microbiota may create an appropriate microbial flora in the ear and can prevent chronic or recurrent infection [90]. Modification of the microbiota may be accomplished through diet management, topical bacteriophage therapy, or the use of topical or oral probiotics [90].

On the other hand, it is crucial that active ingredients are delivered to an infectious site at a therapeutically effective dose for an intended duration to achieve the successful treatment of COE. There are different strategies that can be employed to improve the outcomes of therapy. Several studies utilised biodegradable hydrogels [91], thermosensitive in situ otic gel [92], and nanotechnology [93,94,95] in their otic formulations, many of which have shown improved local retention time for sustained drug release and potentially reduced systemic toxicity. However, many of these studies were limited to either potential human applications or other animal species or were intended to treat ear conditions via intracochlear or intratympanic routes, which would require further studies specific to the treatment of COE.

Finally, another important consideration is owner compliance, which is mainly dependent on the ease of administration and dosing intervals [77]. Developing a new dosing device can be challenging as there are many factors to consider, such as regulatory status, cost, dosing accuracy, repeatability, the viscosity range of the dispensing formulation, and ergonomics. These factors can affect technical feasibility and commercial viability, which should be carefully balanced with achieving drug delivery to the target site at the correct dose for the intended duration of COE treatment. Although there exist many challenges in the development of a new otic formulation for COE, continuing research in the development of novel drug delivery systems, nanoparticles, and application devices may improve treatment options for COE.

## Figures and Tables

**Table 1:** Alternative therapies for the treatment of canine otitis externa.

Therapy	Examples	Study Design	Sample Size	Comparator	Otitis Type	Route of Administration	Duration of Treatment	Outcomes	Evidence Quality Rating	Limitations of the Study	References
Aromatherapy	Volatile essential oil (mixture of sweet almond oil, bergamot oil, lavender oil, tea tree oil, and roman chamomile oil) administered topically	Controlled experimental study; 2-week intervention comparing topical aroma-oil to standard antibiotic therapy	Control: 5 dogs Experimental: 6 dogs	Amoxicillinclavulanic acid; ciprofloxacin; and ketoconazole	Present dogs	Topically to ear canal	Twice daily for two weeks	The treatment group showed a significantly lower bacterial count (*p* < 0.05) than the control	Moderate	A small number of dogs were used. Further studies are required to evaluate the safety and effectiveness of aromatherapy.	[12]
	Oregano oil, thyme oil, carvacrol and thymol	In vitro experimental study evaluating antimicrobial activity of essential oils and phenolic constituents against bacterial and fungal isolates from canine otitis externa	100 isolates	Ampicillin, gentamicin, and amphotericin B	Present	in vitro	NA	MIC_90_: 0.015 to 0.03% for Gram-positive bacteria and *P. mirabilis* MIC_90_: 0.09 to 0.25% for *P. aeruginosa* and *M. pachydermatis*	Moderate	In vivo investigation required	[42]
Acupuncture	Injection of apitoxin in combination with topical aroma oil	Prospective controlled experimental study; 2-week intervention comparing aromatherapy + apipuncture to ketoconazole in dogs with Malassezia-related otitis externa	Control: 5 dogs Experimental: 5 dogs	Ketoconazole	*Malassezia*- related otitis externa	Topical and injection	2 weeks	No hepatotoxicity was observed as compared to the control group (Ketoconazole) and similar antifungal activity	Moderate	Limited samples used for *Malaissezia-related* otitis externa	[14]
Bacteriophages	Topical composition containing 1 × 10^5^ plaque forming units of bacteriophage strains active against *P. aeruginosa*	Prospective uncontrolled clinical trial evaluating single-dose topical bacteriophage therapy for chronic Pseudomonas aeruginosa otitis in dog	Control: 0 Experimental:10 Dogs	None	Chronic *Pseudomonas aeruginosa* otitis	Topical	A single dose of topical preparation, observation after 48 h of administration	Drop in clinical scores after 48 h (signifying improving conditions) against *Pseudomonas aeruginosa* otitis	Low	Small study	[13]
Nutraceuticals	Microcapsules composed of 60–80% of hydrolyzed fish oils, 20–40% minerals and other therapeutic materials (Tea tree oil *Melaleuca alternifolia* 0.00343%, Linden oil *Tilia platyphyllos scapoli et cordata,* 0.0147% Garlic *Allium sativum* L., 0.0245%*, Rosa canina* L., 0.098%, and Zinc, 0.00479%)	Prospective randomized controlled trial; 90-day intervention comparing nutraceutical diet + topical treatment to standard diet + topical treatment in dogs with chronic bilateral otitis externa	Control: 15 dogs Experimental: 15 dogs	Placebo	Chronic bilateral otitis externa	Oral	Once a day for 90 days In addition, all dogs (treatment and control groups) were treated with OTOMAX- 8 drops a day for 7 days	Significantly decreased mean score intensity of chronic bilateral otitis externa over a period of 90 days intervention (*p* < 0.0001)	High	Further studies required with a larger sample and extended observation period.	[3]
Nanomedicine	Silver nanoparticles	In vitro experimental study evaluating dose-dependent antibiofilm activity of silver nanoparticles against *S. pseudintermedius* isolates from dogs with otitis externa	10 isolates	Untreated bacterial isolates	Otitis externa present	in vitro	Microtiter plate and Congo red agar method	Notable antibiofilm activity depends on the dosage (20 and 10 µg/mL) against *Staphylococcus pseudintermedius* (isolated from dogs suffering from otitis externa).	Moderate	Ten strains were insufficient to represent the *S. pseudintermedius* bacterial species, and more work was required to match with in vivo conditions of otitis externa, the study confirmed the inhibition of early biofilm formation for 24 h but did not assess the complete suppression of mature biofilm.	[17]

Evidence Quality rating was based on the following rubric: High quality: Randomized, blinded, adequate sample size. Moderate quality: Controlled but non-randomized, small sample. Low quality: Uncontrolled or very small sample.

**Table 2:** Physicochemical parameters of currently used drugs for COE treatment [56].

Type	Drugs	Molecular Weight (g/mol)	XLogP3	PubChem Compound ID
Antibacterial	Bacitracin zinc	1486.1	−4.1 *	70687193
	Fusidic acid diethanolamine	621.8	5.5 *	46174083
	Enrofloxacin	359.4	−0.2	71188
	Florfenicol	358.2	0.8	114811
	Framycetin sulfate	712.7	−9.0 *	197162
	Gentamicin sulfate	575.7	−4.1 *	6419933
	Marbofloxacin	362.4	−0.5	60651
	Orbifloxacin	395.4	0.9 ^a^	60605
	Polymyxin B sulfate	1301.6	Not applicable (large cyclic peptide, no reported LogP)	56842110
Antifungal	Clotrimazole	344.8	5	2812
	Ketoconazole	531.4	4.3	47576
	Miconazole nitrate	479.1	5.3 *	68553
	Nystatin	926.1	−0.2 ^a^	6433272
	Posaconazole	700.8	4.6	468595
	Terbinafine hydrochloride	327.9	5.6 *	5282481
Steroid	Betamethasone valerate	476.6	3.6	16533
	Dexamethasone acetate	434.5	2.8	236702
	Hydrocortisone aceponate	460.6	3.3	68921
	Mometasone furoate	521.4	3.9	441336
	Prednisolone acetate	402.5	2.4	5834
	Triamcinolone acetonide	434.5	2.5	6436

^a^ XLogP3-AA (log P calculated by the atom-additive (AA) method in the XLogP3 model). * Where a PubChem-listed XLogP3 value was not available for a particular salt form, logP for the parent free base or core structure was listed.
